# Ultrafast optical observation of spin-pumping induced dynamic exchange coupling in ferromagnetic semiconductor/metal bilayer

**DOI:** 10.1038/s41598-022-19378-z

**Published:** 2022-11-22

**Authors:** X. Liu, P. Liu, H. C. Yuan, J. Y. Shi, H. L. Wang, S. H. Nie, F. Jin, Z. Zheng, X. Z. Yu, J. H. Zhao, H. B. Zhao, G. Lüpke

**Affiliations:** 1 Department of Applied Science, College of William and Mary, 251 Jamestown Road, Williamsburg, VA 23187 USA; 2 Department of Optical Science and Engineering, Key Laboratory of Micro and Nano Photonic Structures (Ministry of Education), Shanghai Ultra-Precision Optical Manufacturing Engineering Research Center, Fudan University, Shanghai, 200433 China; 3 State Key Laboratory of Supperlattices and Microstructures, Institute of Semiconductors, Chinese Academy of Sciences, Beijing, 100083 China

**Keywords:** Condensed-matter physics, Materials for devices, Ferromagnetism, Spintronics, Surfaces, interfaces and thin films

## Abstract

Spin angular momentum transfer in magnetic bilayers offers the possibility of ultrafast and low-loss operation for next-generation spintronic devices. We report the field- and temperature- dependent measurements on the magnetization precessions in Co_2_FeAl/(Ga,Mn)As by time-resolved magneto-optical Kerr effect. Analysis of the effective Gilbert damping and phase shift indicates a clear signature of an enhanced dynamic exchange coupling between the two ferromagnetic (FM) layers due to the reinforced spin pumping at resonance. The temperature dependence of the dynamic exchange-coupling reveals a primary contribution from the ferromagnetism in (Ga,Mn)As.

## Introduction

There has been growing interest in the ultrafast optical manipulation of magnetic dynamics in ferromagnetic heterostructures due to its potential applications in advanced functional spintronic devices. The spin-pumping (SP) effect, in which a spin-precessing ferromagnetic layer transfers its angular momentum into another layer by a chargeless spin current, brings a new mechanism for spin controlling and hence plays an important role in the design of future spintronic devices^1^. Since Heinrich et al. first reported the spin-pumping effect as increased damping of the source layer in ferromagnetic resonance (FMR) experiments^2^ and a few FMR experiments on SP effect have been performed on transition-metal multilayers^3–7^, topological insulators^8,9^ and semiconductors^10–12^. In addition, Danilov et al. demonstrated that the mutual SP effect modifies the precession dynamics in a pseudo spin-valve where magnetization precessions are excited simultaneously in two FM layers by femtosecond laser pulses^13^. However, no SP effect has ever been observed yet for the heterostructure of a Heusler alloy and a ferromagnetic (FM) semiconductor. Importantly, the hard and soft ferromagnetic phases in such materials can potentially exhibit a dynamic exchange coupling that is completely independent of the static exchange coupling due to spin pumping. This could offer a possibility of ultrafast low-power control of spin current for next-generation spintronic devices.

In this study, we investigate the magnetization precession dynamics of the Heusler alloy Co_2_FeAl/FM semiconductor (Ga,Mn)As heterostructure as a function of applied field and temperature by time-resolved magneto-optical Kerr effect (TRMOKE). Analysis of the field-dependent effective Gilbert damping indicates a clear signature of the enhanced dynamic exchange coupling between the two FM layers due to a reinforced spin pumping. In addition, curvatures of the phase shift as a function of applied field elucidate the dynamic exchange-coupling model where the counter-precessing precessions are damped significantly at the resonant frequency of the two FM layers. The magnetization precession in the Co_2_FeAl layer transfers a pure spin current directly into the ferromagnetic semiconductor (Ga,Mn)As layer without a nonmagnetic metal spacer. On the other hand, the temperature-dependent results manifest a strong contribution from the ferromagnetism of (Ga,Mn)As to the dynamic exchange-coupling effect. These results provide valuable insight into the topic of dynamic exchange coupling and the detection of spin current. Furthermore, they suggest a new pathway of ultrafast spin manipulation in metal/semiconductor bilayer systems at low power and therefore promote the development and design of future spintronic devices.

The Co_2_FeAl/(Ga,Mn)As bilayer sample is grown on GaAs (001) substrates by molecular-beam epitaxy (MBE). The thickness of Co_2_FeAl and Ga_1-x_Mn_x_As (*x* = 0.07) layer is 10 nm and 150 nm, respectively. The sample is capped with 2-nm thick Al layer to avoid oxidation and contamination. The hard FM Co_2_FeAl layer with a 10-nm thickness shows an in-plane uniaxial magnetic anisotropy with an easy axis along the [110] direction (Figure S1, see Supplementary), whereas the soft FM (Ga,Mn)As layer with a 150-nm thickness shows an easy axis along the^1–10^ direction at *T* = 15 K, revealed by the minor loop (Figure S1, see Supplementary). The static magnetic properties characterized in this study are similar to those of the Co_2_FeAl (3 nm)/(Ga,Mn)As (150 nm) heterostructures reported by Nie et al.^14^ Reflection high-energy electron diffraction (RHEED) patterns, high-resolution double-crystal x-ray diffraction (DCXRD) measurements, and high-resolution cross-sectional transmission electron microscopy (HRTEM) reveal high-quality, single-crystalline, epitaxial growth of the Co_2_FeAl and (Ga,Mn)As thin films^13^. At low temperatures (*T* < *T*_*c*_ = 50 K), a ferromagnetic alignment of local Mn moments in the (Ga,Mn)As layer is expected, whereas at high temperatures (*T* > *T*_*c*_ = 50 K) the Mn ions extending a few nanometers from the interface remain spin-polarized due to the ferromagnetic proximity effect^14^.

**Figure 1:**
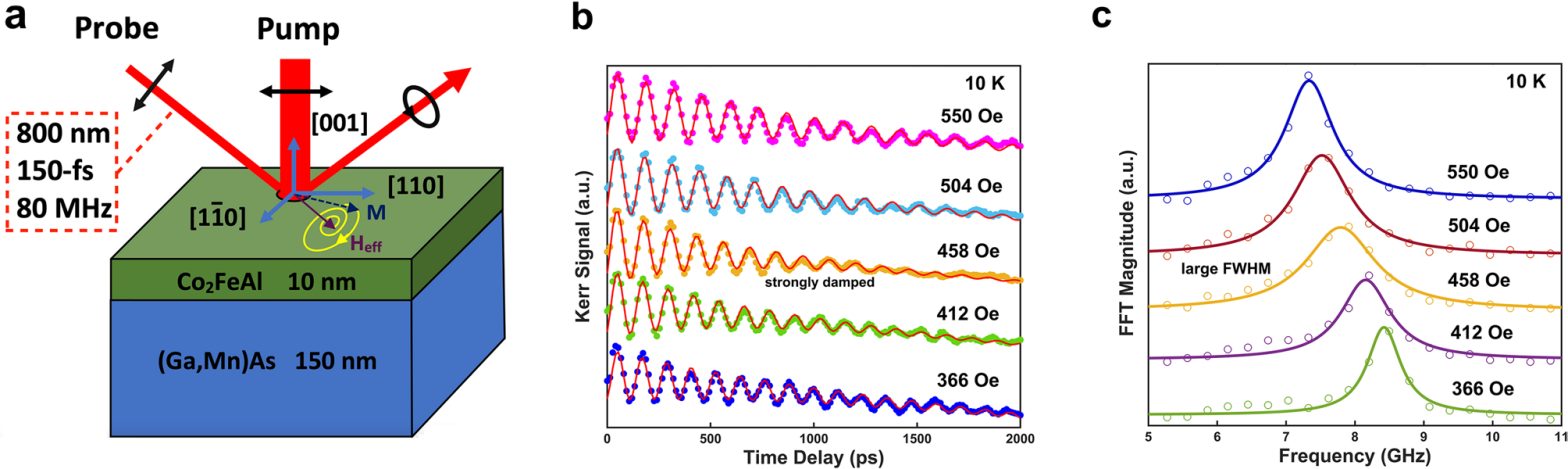
(**a**) Schematic of TRMOKE measurement geometry, depicting the structure of the sample and the magnetization *M* precessing around the effective field *H*_*eff*_ in Co_2_FeAl/(Ga,Mn)As bilayer in a canted magnetization configuration with *H* applied along hard axis^1–10^. (**b**) TRMOKE data from Co_2_FeAl/(Ga,Mn)As bilayer under the different intensity of the applied field. (**c**) FFT analysis for magnetization precession frequency under the different intensities of the applied field, where the solid lines represent the FFT peaks fitted by Lorentz functions.

Figure 1a shows the experimental geometry of TRMOKE measurements. Field-dependent TRMOKE measurements are performed on the Co_2_FeAl/(Ga,Mn)As bilayer sample from 7 to 300 K utilizing 100-fs pump and probe pulses at 800 nm wavelength with a repetition rate of 80 MHz. The external magnetic field is set along the easy-hard axis [100] of the Co_2_FeAl layer and the pump fluence is set at *I* = 5 µJ/cm^2^. The probe pulses utilize the balanced detection technique with a half-wave plate and Wollaston prism to investigate the transient magnetic state change along longitudinal and polar directions. Figure 1b displays the TRMOKE data at 10 K with an in-plane magnetic field scanning from 366 to 550 Oe. The precession signals can be well fitted by a damped-harmonic function with a linear background:$\theta_{k} = a_{0} + b_{0} t + A \times \exp \left( { - \frac{t}{\tau }} \right)\sin \left( {2\pi ft + \varphi_{0} } \right)$, where $a_{0} + b_{0} t$ represents the linear approximation of the background signal related to a slow recovery of the magnetization , *A* is the precession amplitude, $\tau$ is the relaxation time, *f* is the precession frequency and $\varphi_{0}$ is the phase. The magnetization precession decays with different relaxation times, with the fastest decay at 458 Oe. This indicates that a dynamic exchange coupling may occur between the magnetization precession in the Co_2_FeAl layer and the (Ga,Mn)As layer. Here, only one frequency can be extracted from FFT analyses, as seen in Fig. 1c, coherent spin precession of (Ga,Mn)As decays fast and thus vanishes very shortly.

The magnetization precession in the Co_2_FeAl/(Ga,Mn)As bilayer system is described by the following modified Landau-Lifshitz-Gilbert (LLG) equation with an additional spin-torque term:

$$\frac{dm}{{dt}} = - \gamma m \times H_{{{\text{eff}}}} + \alpha_{0} m \times \frac{dm}{{dt}} + \alpha_{sp} \left( {m \times \frac{dm}{{dt}} - m^{\prime} \times \frac{{dm^{\prime}}}{dt}} \right)$$

where $m$ is the magnetization direction of the Co_2_FeAl layer, $\gamma$ is the gyromagnetic ratio, $\alpha_{0}$ is the intrinsic Gilbert damping constant, and $H_{{{\text{eff}}}}$ is the effective magnetic field in the Co_2_FeAl layer including the external magnetic field, the demagnetization field, the anisotropy field, and the exchange-coupling field. The last term describes the spin torque which acts on both layers as a bidirectional effect, in which $\alpha_{sp}$ represents the contribution of spin pumping to the damping and $m^{\prime}$ denotes the magnetization of (Ga,Mn)As. Then, the effective Gilbert damping can be obtained from the relaxation time $\tau$, using^15^

$$\alpha = 2/\left[ {\tau \gamma \left( {H_{a} + H_{b} } \right)} \right]$$

where $H_{a}$ and $H_{b}$ are determined by fitting the magnetization precession frequency and anisotropy energy terms (see Supplementary), which includes the out-of-plane, in-plane uniaxial, crystalline cubic, unidirectional and rotatable magnetic anisotropies.

**Figure 2:**
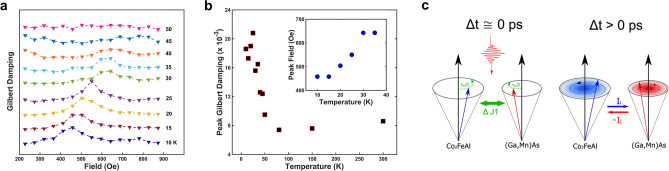
(**a**) Effective Gilbert damping constant as a function of externally applied field at different temperatures from 10 to 50 K. (**b**) Red rectangular solids denote the peak (or strongest if not peak) Gilbert damping from 10 to 50 K. Blue circle solids are the corresponding fields as a function of temperature. (**c**) Illustration of photo-excited exchange-coupling torque and spin-pumping generated dynamic exchange-coupling mode for damping.

Figure 2a shows the temperature-dependent Gilbert damping as a function of the external field. At *T* = 10 K, the damping of the magnetization precession is most pronounced with an external field *H* = 458 Oe. Below the Curie temperature (*T*_c_ = 50 K) of (Ga,Mn)As, the damping peak first shifts from 450 to 650 Oe with the temperature increasing from 10 to 35 K, as summarized in Fig. 2b insert, and then gradually becomes inconspicuous and finally disappears at *T* = *T*_c_. Such a temperature dependency clearly shows that the damping peak only exists when the ferromagnetism of (Ga,Mn)As is well-developed. Meanwhile, the strongest Gilbert damping extracted across all the fields as a function of temperature (Fig. 2b) shows a transition temperature close to *T*_c_. This manifests the crucial role of spontaneous (Ga,Mn)As magnetization in the damping of magnetization precession of Co_2_FeAl.

The ultrafast pump excitation causes a transient modification of exchange coupling, ^16^ which induces a dynamic exchange-coupling torque acting on both Fe(Co) spins and Mn spins. In such a case, as shown in Fig. 2c, the magnetizations of both FM layers are suddenly pulled towards each other and start to precess with opposite angular momentum along their own equilibrium directions. At the resonance, i.e., *f*_CFA_ = *f*_GMA_, the precessing magnetization of Co_2_FeAl “pumps” a spin current *I*_*i*_ directly into the (Ga,Mn)As layer, which exerts a torque onto the (Ga,Mn)As magnetization and thereby counteracts its precession. Meanwhile, this spin current *I*_*i*_ carries an outflow of angular momentum from the Co_2_FeAl layer and leads to damping to its magnetization precession. In other words, the spin current reinforces the *M* damping for both FM layers at the resonance. Technically, there should also be a spin current *I*_*j*_ injecting into the Co_2_FeAl layer from the magnetization precession of (Ga,Mn)As^2^. However, such a spin current should be much smaller than that from the Co_2_FeAl layer.

**Figure 3:**
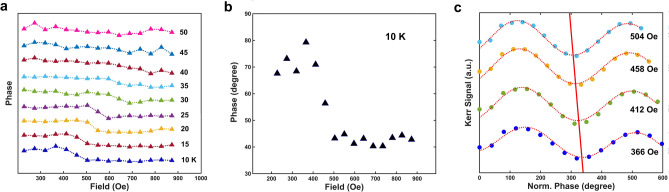
Phase of magnetization precession as a function of externally applied field at different temperatures from 10 to 50 K (**a**), and zoomed specifically at 10 K (**b**). (**c**) TRMOKE data as a function of normalized precession phase under different applied fields. The red straight line is a guideline of the phase shift.

In addition to the discussion on damping, the dynamic exchange coupling between the two FM layers can also be evinced by the field-dependency of the precession phase. Figure 3a shows that from 10 to 35 K, the phase drops down dramatically around certain field windows that correspond to the Gilbert damping peaks, which move to higher field ranges as temperature increases. When *T* > 30 K, the dramatic phase shift becomes less contrastive and then completely disappears when *T* = *T*_c_. We notice that similar features of phase-shifting are reported in the FMR experiments on similar hard/soft FM systems^17,18^. The observed 30° ~ 40° phase shift at 10 K, as shown in Fig. 3c, is comparable with those of the dynamic exchange-coupling spin-valve structures^8,17–20^.

In summary, we have studied the dynamics of the magnetization precession of Co_2_FeAl/(Ga,Mn)As heterostructure as a function of applied field and temperature. The pronounced peaks in effective Gilbert damping of Co_2_FeAl magnetization precession reveal the enhanced dynamic exchange coupling between the two FM layers due to spin pumping. The corresponding field-dependency of phase shift, which corresponds to that of the Gilbert damping, confirms the counter-precessing exchange-coupled model where both precessions are damped significantly at the resonance. In addition, the temperature-dependent results manifest a strong contribution from the ferromagnetism in (Ga,Mn)As to the dynamic exchange-coupling effect. These results provide valuable insight into the topic of dynamic exchange coupling and the detection of spin current. Mover, they suggest a novel route of ultrafast low-power spin manipulation in metal/semiconductor bilayer system and hence promote the research of the future spintronic devices.

## Methods

### MOKE experiments

The magnetization of the exchange-coupled Co_2_FeAl/(Ga,Mn)As bilayer is measured using a longitudinal MOKE setup. The sample is illuminated with p-polarized light and the reflected s-polarized light is detected with a photodiode. The magnetic field is applied along the in-plane [110] or [−110] crystallographic directions. The measurements are conducted from 5 K to above room temperature.

### TRMOKE experiments

For the pump-probe TRMOKE measurements, a Ti:sapphire oscillator laser system is employed, which produces 150-fs pulses at 800-nm wavelength with a repetition rate of 80 MHz. The probe (pump) fluence is fixed at ~ 0.5 (5) µJ/cm^2^. The probe pulses (*λ* = 800 nm) use the balanced detection approach with a half-wave plate and Wollaston prism to probe the transient magnetic state change along longitudinal and polar directions. The measurements are conducted from 5 K to above room temperature.

## Supplementary Information

Supplementary Information.

## Data Availability

The data that support the findings of this study are available from the corresponding author upon reasonable request.
