# Supplementary material for: Ultrafast optical observation of spin-pumping induced dynamic exchange coupling in ferromagnetic semiconductor/metal bilayer
Source: Sci Rep. 2022 Nov 22;12:20093. doi: 10.1038/s41598-022-19378-z (PMC9684537; doi:10.1038/s41598-022-19378-z)
Supplement: Supplementary file 1 — Supplementary Information. [file 41598_2022_19378_MOESM1_ESM.docx]

**Supplementary:** Ultrafast optical observation of spin-pumping induced dynamic exchange coupling in ferromagnetic semiconductor/metal bilayer

X. Liu^1,#^, P. Liu^1,#^, H. C. Yuan^2^, J. Y. Shi^2^, H. L. Wang^3^, S. H. Nie^3^, F. Jin^2^, Z. Zheng^2^, X. Z. Yu^3^, J. H. Zhao3^,^ *, H. B. Zhao2,*, and G. Lüpke1,*

1 Department of Applied Science, College of William and Mary, 251 Jamestown Road, Williamsburg, Virginia 23187, USA

2 Key Laboratory of Micro and Nano Photonic Structures (Ministry of Education), Shanghai Ultra-precision Optical Manufacturing Engineering Research Center, Department of Optical Science and Engineering, Fudan University, Shanghai, 200433, China

3 State Key Laboratory of Supperlattices and Microstructures, Institute of Semiconductors, Chinese Academy of Sciences, Beijing 100083, China

*email: [jhzhao@red.semi.ac.cn](mailto:jhzhao@red.semi.ac.cn); [hbzhao@fudan.edu.cn](mailto:hbzhao@fudan.edu.cn); [gxluep@wm.edu](mailto:gxluep@wm.edu)

^#^ X. Liu and P. Liu contributed equally to the work.

**Figure S1: Results of SQUID measurements**

**
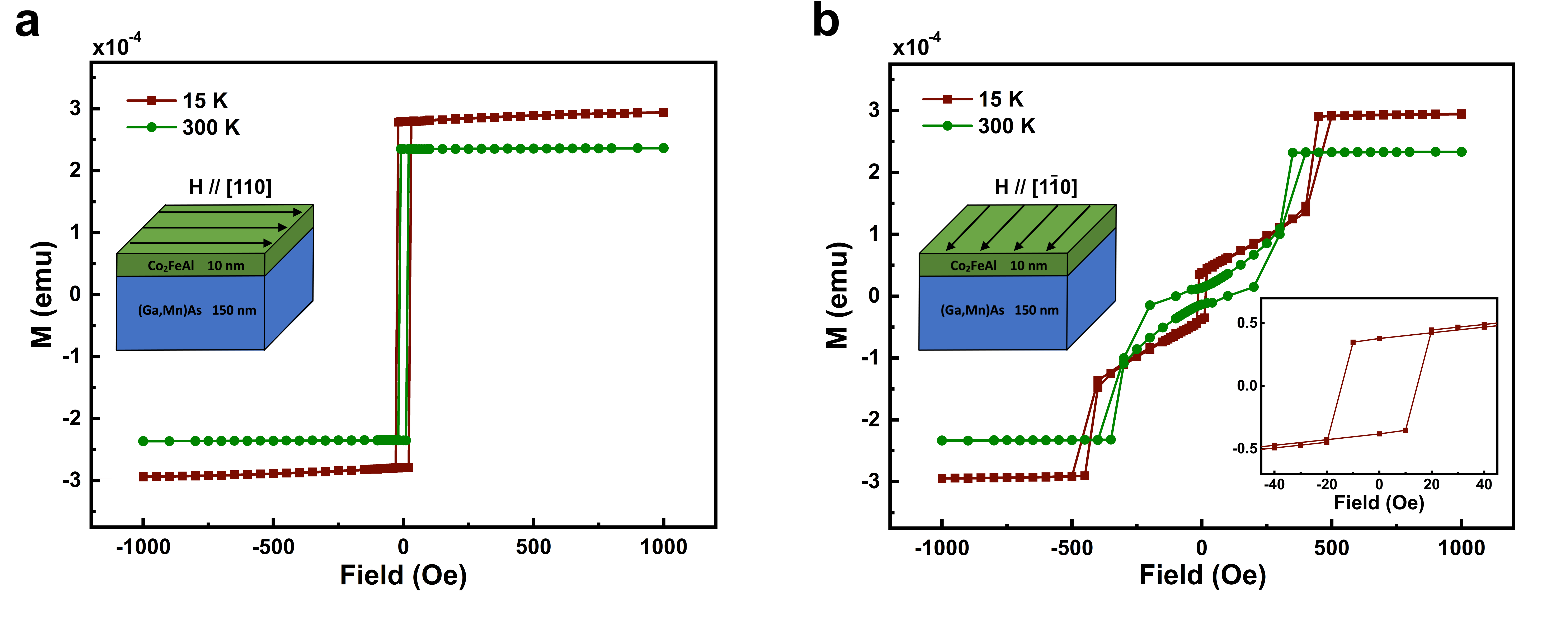
**

**Figure S1.** (a) SQUID measurement in Co_2_FeAl/(Ga,Mn)As bilayer structure along the easy axis [110] direction. (b) SQUID measurement in Co_2_FeAl/(Ga,Mn)As bilayer structure along the hard axis [1-10] direction. The inset shows the close-up around zero magnetic field at 15 K.

**Equation S1: Magnetic free energy (*E*)**

$$E=-M_{s}Hcos\left( \varphi_{M}-\varphi_{H} \right)-\left( 2\pi M_{s}^{2}+K_{\perp} \right){sin}^{2}\varphi_{\perp}+K_{u}{sin}^{2}\varphi_{M}+\frac{K_{1}}{4}{sin}^{2}2\varphi_{M}$$

$+K_{ud}sin\left( \varphi_{M}-\varphi_{ud} \right)+K_{ra}{cos}^{2}\left( \varphi_{M}-\varphi_{H} \right)+J_{1}{M_{s}M}_{s}^{'}cos\theta$ (S1)

The last term $J_{ex}=J_{1}{M_{s}M}_{s}^{'}cos\theta$ represents the contribution from the exchange coupling effect, where $M_{s}$ is the saturation magnetization in the Co_2_FeAl layer and $M_{s}^{'}$ is the saturation magnetization in the (Ga,Mn)As layer. $J_{1}$ is the exchange-coupling stiffness and *θ* is the angle between the magnetization directions in Co_2_FeAl and (Ga,Mn)As. *H* is the applied field. $\varphi_{M}$ , $\varphi_{H}$, $\varphi_{\perp}$ and $\varphi_{ud}$ are the angles of magnetization, applied field, perpendicular and unidirectional anisotropy, respectively. $K_{\perp}$, $K_{u}$, $K_{1}$, $K_{ud}$ and $K_{ra}$ are the out-of-plane, in-plane uniaxial, crystalline cubic, unidirectional and rotatable magnetic anisotropies, respectively.

**Equation S2: Spin precession frequency (*f*)**

$f= \frac{\gamma}{2\pi}{(H_{a}\cdot H_{b})}^{\frac{1}{2}}$ (S2)

where

$$H_{a}=Hcos\left( \varphi_{M}-\varphi_{H} \right)+H_{u}cos\left( 2\varphi_{M} \right)+H_{1}cos\left( 4\varphi_{M} \right)-\frac{H_{ud}}{2}sin\left( \varphi_{M}-\varphi_{ud} \right)-H_{ra}cos2\left( \varphi_{M}-\varphi_{H} \right)-J_{1}M_{s}^{'}cos\theta$$

$$H_{b}=Hcos\left( \varphi_{M}-\varphi_{H} \right)+4\pi M_{eff} {- H}_{u}{sin}^{2}\varphi_{M}+\frac{H_{1}}{4}\left( 3+cos\left( 4\varphi_{M} \right) \right)-\frac{H_{ud}}{2}sin\left( \varphi_{M}-\varphi_{ud} \right)-H_{ra}{cos}^{2}\left( \varphi_{M}-\varphi_{H} \right)-J_{1}M_{s}^{'}cos\theta$$

with $M_{eff}=M_{s}+\frac{K_{\perp}}{2\pi M_{s}}$ , $H_{u}=\frac{2K_{u}}{M_{s}}$, $H_{1}=\frac{2K_{1}}{M_{s}}$, $H_{ud}=\frac{2K_{ud}}{M_{s}}$, $H_{ra}=\frac{2K_{ra}}{M_{s}}$ and gyromagnetic ratio *γ* = 1.76×10^7^ Hz/Oe.

**Note 1: Extraction of Gilbert damping constants**

The magnetic anisotropy fields are obtained by analyzing the field dependence of the magnetization precession frequency from temperature T = 10 K to 50 K. First, by minimizing the magnetic free energy *E* (Equation S1), the angle of magnetization directions is determined as a function of external field for a certain range of anisotropy fields. Then we can obtain the value of magnetic anisotropy fields by fitting the precession frequency *f* with Equation S2. With the relaxation time τ derived by fitting the damped-harmonic function, we then obtain the effective Gilbert damping constant by $\alpha=2/\left[ \tau\gamma\left( H_{a}+H_{b} \right) \right]$.
